# Additive manufacturing of an ultrastrong, deformable Al alloy with nanoscale intermetallics

**DOI:** 10.1038/s41467-024-48693-4

**Published:** 2024-06-15

**Authors:** Anyu Shang, Benjamin Stegman, Kenyi Choy, Tongjun Niu, Chao Shen, Zhongxia Shang, Xuanyu Sheng, Jack Lopez, Luke Hoppenrath, Bohua Peter Zhang, Haiyan Wang, Pascal Bellon, Xinghang Zhang

**Affiliations:** 1 School of Materials Engineering, Purdue University, West Lafayette, IN 47907 USA; 2 Department of Materials Science and Engineering, University of Illinois Urbana-Champaign, Champaign, IL 61801 USA; 3 Los Alamos National Lab, Albuquerque, NM 87545 USA

**Keywords:** Metals and alloys, Mechanical properties, Mechanical engineering

## Abstract

Light-weight, high-strength, aluminum (Al) alloys have widespread industrial applications. However, most commercially available high-strength Al alloys, like AA 7075, are not suitable for additive manufacturing due to their high susceptibility to solidification cracking. In this work, a custom Al alloy Al_92_Ti_2_Fe_2_Co_2_Ni_2_ is fabricated by selective laser melting. Heterogeneous nanoscale medium-entropy intermetallic lamella form in the as-printed Al alloy. Macroscale compression tests reveal a combination of high strength, over 700 MPa, and prominent plastic deformability. Micropillar compression tests display significant back stress in all regions, and certain regions have flow stresses exceeding 900 MPa. Post-deformation analyses reveal that, in addition to abundant dislocation activities in Al matrix, complex dislocation structures and stacking faults form in monoclinic Al_9_Co_2_ type brittle intermetallics. This study shows that proper introduction of heterogeneous microstructures and nanoscale medium entropy intermetallics offer an alternative solution to the design of ultrastrong, deformable Al alloys via additive manufacturing.

## Abstract (web summary)

Commercial high-strength Al alloys often suffer from cracking during additive manufacturing. Here, the authors present an additively manufactured, a strong and plastically deformable Al alloy with heterogeneous nanoscale intermetallics.

## Introduction

Aluminum (Al) alloys are widely utilized as structural materials in aerospace and automobile industries^1,2^. To fulfill the complex geometrical constraints for industrial applications, selective laser melting (SLM) has been increasingly used to fabricate parts of Al alloys, offering a high level of design flexibility. Most existing studies have been conducted mainly for near-eutectic Al-Si and Al-Si-Mg alloys^3^. These alloys exhibit medium strength but great hot-tearing resistance, making them good candidates for 3D printing^2,4,5^. In contrast, high-strength Al alloys, such as AA 6061^6^ and AA 7075^7^, are inherently susceptible to hot cracking during additive manufacturing process.

One method to alleviate hot cracking during additive manufacturing of high-strength Al alloys is to introduce fine and hard particles^5,8^. These particles can be introduced via external inoculation, e.g. TiN^9,10^, TiC^11–13^, TiB_2_^14–17^ or aging, e.g. Al_3_Zr^18–20^, Al_3_Sc^21,22^, Al_2_Cu^23,24^, and can strengthen the Al alloy by impeding dislocation movements. Meanwhile, these particles promote heterogeneous nucleation, and break down columnar grains where intergranular cracks are prone to initiate and propagate^20^. In spite of these studies, the highest strength achieved in additively manufactured (AM) Al alloys remains in the range of 300–500 MPa^2^. There is scattered success in producing high strength Al alloys via severe plastic deformation^25^, such as high-pressure torsion and accumulative roll-bonding, or cryo-milling followed by powder consolidation^26^. The high strength in these cases arises from significant grain refinement to nanoscales. Ultra-strong AM Al alloys with high flow strength and deformability remain to be discovered.

Transition metal (TM) intermetallics, such as Al-Fe, Al-Co and Al-Ni are largely avoided in AM Al alloys as prior experience in casting shows that the addition of TM elements often leads to large and brittle intermetallics^27,28^. These intermetallics, such as Al_9_Co_2_, Al_13_Fe_4_ have crystal structures with low symmetry (monoclinic) and thus are known to be brittle materials at room temperature. Recent studies show the addition of a small amount of Fe or Ni, can improve the mechanical strength of Al alloys, but the plastic deformability and deformation mechanisms remain unknown^28^.

In this work, several transition metal elements including Co, Fe, Ni and Ti are selected to produce intermetallics-strengthened AM Al alloys. Colonies of nanoscale intermetallics lamellae aggregate into fine rosettes and give rise to a high strength, exceeding 700 MPa, with prominent plastic deformability under compression. The heterogeneous microstructure also introduces significant back stress. Complex dislocation structures and stacking faults are present in the sandwiched monoclinic brittle Al_9_(Fe,Co,Ni)_2_ phase. This study demonstrates an effective strategy to develop ultrahigh-strength AM Al alloys via nanoscale laminated deformable intermetallics.

## Results

### Microstructural characterization

**Fig. 1:**
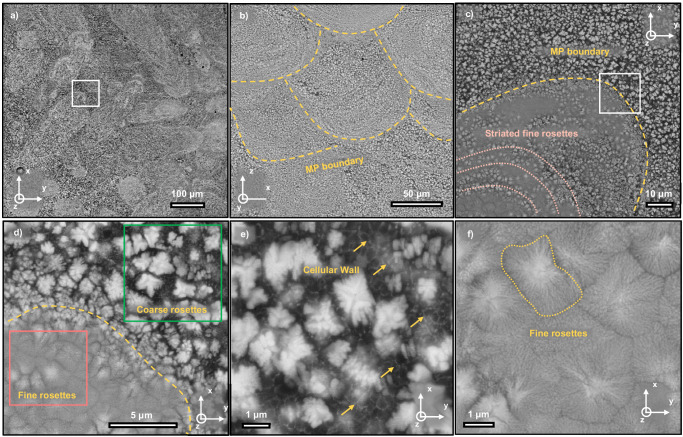
Back scattered scanning electron microscopy (SEM) images showing overview microstructure of the as-printed Al_92_Ti_2_Fe_2_Co_2_Ni_2_ alloy with 300 W laser. **a** The horizontal (XY) view. The region outlined in the white box is enlarged in (**c**). **b** The vertical (XZ) planes. Z is the build direction. Yellow dash lines outline the melt-pool (MP) boundaries. **c** Micrograph of a representative melt pool with the outlined boundary and striated fine rosettes. The region outlined in the white box is magnified in (**d**). **d** The microstructure across the melt pool boundaries showing distinctive features in the coarse rosettes region and fine rosettes region. The regions outlined in the green and pink boxes are enlarged in (**e**, **f**) for coarse rosettes and fine rosettes, respectively. **e** A micrograph of the coarse rosettes region with yellow arrows indicating cellular precipitates. **f** A micrograph of the fine rosettes region showing the densely packed fine rosettes marked by yellow dotted lines with limited content of Al.

Back scattered scanning electron microscopy (SEM) images reveal the microstructure of the as-printed Al_92_Ti_2_Fe_2_Co_2_Ni_2_ fabricated with 300 W laser power in Fig. 1. Morphologies of inter-woven laser tracks and inverse-parabolic melt pool cross sections typical in SLM-processed alloys are evident on horizontal XY plane (Fig. 1a) and vertical XZ plane (Fig. 1b), where Z axis is the build direction. Melt pools outlined by yellow dash lines are around 120 µm in width and 80 µm in depth, with some inherent variation due to layer rotations. Figure 1c, d show a gradient heterogeneous microstructure in the melt pools. Colonies of layered aggregates (referred to as rosettes) shown in light contrast are intermingled with the Al rich matrix (in dark contrast). Rosettes with finer laminate spacing (fine rosettes) are the dominant features near the melt pool boundaries, while rosettes with thicker lamellae (coarse rosettes) are abundant in the melt pool center. In addition, there are also some fine rosettes in the melt pool center arranged in a striated fashion outlined in pink. High-magnification SEM micrographs in Fig. 1e, f show that the melt pool center consists of coarse rosette region (36 vol.%), nanoscale cellular precipitates (4 vol.%) denoted by yellow arrows and Al rich matrix (60 vol.%). In contrast, near the melt pool boundaries, fine rosettes (97 vol.%) are separated by thin layers of Al matrix (3 vol.%).

**Fig. 2:**
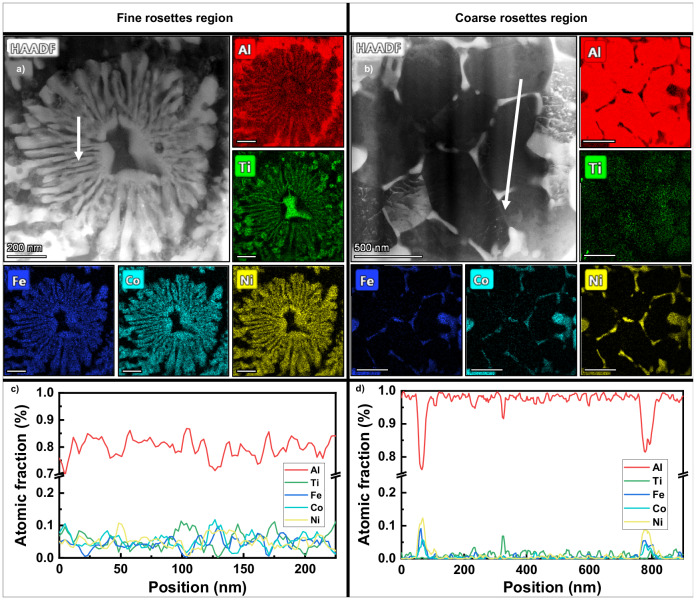
Morphology and compositions of nanoscale intermetallics. High-angle annular dark-field scanning transmission electron microscopy (HAADF-STEM) images showing representative morphologies of (**a**) fine rosettes region and (**b**) coarse rosettes region and corresponding energy-dispersive x-ray spectroscopy (EDS) composition maps of various elements. The line scans in (**c**, **d**) showing relevant phase constituents following the white arrows in (**a**, **b**), respectively. The fine rosettes have an Al_3_Ti core with alternate lamellae composed of Al_3_Ti and Al_9_(Fe,Co,Ni)_2_. The cellular precipitates in coarse rosettes region are composed of Al_9_(Fe,Co,Ni)_2_.

Representative fine rosettes and cellular precipitates in coarse rosette region were characterized by scanning transmission electron microscopy (STEM) and energy dispersive x-ray spectroscopy (EDS) in Fig. 2. The fine rosettes as shown in Fig. 2a, c have Al_3_Ti cores surrounded by two alternating intermetallics laminates, Al_3_Ti and medium entropy Al_9_(Fe,Co,Ni)_2_ intermetallics, with a laminate thickness of 20–60 nm. The Al_3_Ti layers are thinner than Al_9_(Fe,Co,Ni)_2_ in the laminates. The chemical compositions for coarse rosettes are nearly identical to fine rosettes with a laminate thickness of 150–300 nm. The coarse rosette region also contains cellular boundaries enriched in Al_9_(Fe,Co,Ni)_2_ as shown in Fig. 2b, d.

**Fig. 3:**
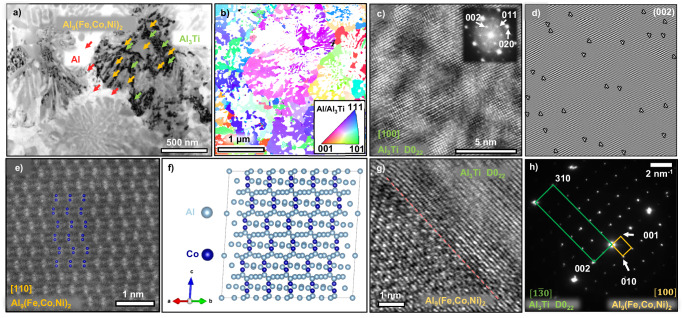
TEM characterizations of the as-printed Al_92_Ti_2_Fe_2_Co_2_Ni_2_. **a** An overview transmission electron microscopy bright field (TEM-BF) image showing three major phases in the alloy, Al matrix (red arrows), Al_9_(Fe,Co,Ni)_2_ (orange arrows), and Al_3_Ti (green arrows). **b** Inverse pole figure mapping of Al/Al_3_Ti phases by ASTAR (nano-EBSD based on high-precision electron diffraction patterns). **c** High-resolution TEM (HRTEM) with the corresponding Fast Fourier Transform (FFT) of the image of Al_3_Ti intermetallic showing its highly defective nature. **d** An inverse FFT of the image with (002) plane filtered indicating the presence of abundant dislocations in the nanoscale Al_3_Ti intermetallics. **e** High-resolution STEM (HRSTEM) showing the atomic arrangement of Al_9_(Fe,Co,Ni)_2_ intermetallic compound superimposed with cobalt atoms. **f** Identical structures obtained from VESTA^35^ crystallographic visualization of its prototype Al_9_Co_2_ with monolithic crystal structure. **g** HRTEM micrograph of the interface between two genres of intermetallics, and (**h**) the corresponding selected area electron diffraction (SAED) pattern indicating the orientation relationships.

Detailed microstructure examinations of the same (300 W) specimen using TEM and STEM are summarized in Fig. 3. The bright-field (BF) TEM image in Fig. 3a shows the coexistence of three major phases, Al matrix (red arrows), Al_9_(Fe,Co,Ni)_2_ (orange arrows), and Al_3_Ti (green arrows). X-Ray diffraction (XRD) pattern (Supplementary Fig. 1) and selected area electron diffraction (SAED) of the TEM image (Supplementary Fig. 3) suggest the Al_3_Ti exists mainly in D0_22_ phase (space group I4/mmm, a = 0.384 nm, c = 0.860 nm^29^) and partially in L1_2_ phase (space group ${{{{{\rm{Pm}}}}}}\bar{3}{{{{{\rm{m}}}}}}$, a = 0.398 nm^30^), and Al_9_(Fe,Co,Ni)_2_ has monoclinic structure with the prototype of Al_9_Co_2_ (space group P2_1_/c, a = 0.622 nm, b = 0.629 nm, c = 0.856 nm, β = 94.8° ^31^) or Al_9_FeNi^32^. Besides, it could be seen that plenty of defects exist in Al_3_Ti (Fig. 3a & Supplementary Fig. 3). The inverse pole figure (IPF) map in Fig. 3b acquired from high-resolution ASTAR orientation mapping by high-precision electron diffraction recognition^33,34^ demonstrates the crystallographic orientation of Al and Al_3_Ti. The polycrystalline composite has colonies with dimension of ~ 1 μm. High-resolution TEM (HRTEM) image (Fig. 3c) along Al_3_Ti D0_22_ [100] zone axis demonstrates the lattice distortion induced by the 2 nm scale scattered patches. An inverse Fast Fourier Transform (FFT) image based on (002) diffraction shows plenty of dislocations residing in the disordered lattice patches as shown in Fig. 3d. In comparison, the Al_9_(Fe,Co,Ni)_2_ phase possesses few defects as shown in Fig. 3a. High-resolution STEM (HRSTEM) (Fig. 3e) shows the atomic arrangements of the medium-entropy Al_9_(Fe,Co,Ni)_2_ along [110] zone axis with blue dots representing sites of Co atoms in its prototype. The micrograph is consistent with the simulated 3 × 3 × 3 cells by VESTA^35^ (Fig. 3f). It’s difficult to distinguish Fe, Co, Ni atoms in the current HRSTEM micrograph due to their close proximity in atomic number. Figure 3g depicts the typical well-defined interface between Al_9_(Fe,Co,Ni)_2_ and Al_3_Ti. The SAED pattern (Fig. 3h) illustrates the crystallographic orientation relationships between these two phases, where ${\left[1\bar{3}0\right]}_{{{{{{\rm{A}}}}}}{{{{{{\rm{l}}}}}}}_{3}{{{{{\rm{Ti}}}}}}}$ // ${\left[100\right]}_{{{{{{\rm{A}}}}}}{{{{{{\rm{l}}}}}}}_{9}{\left({{{{{\rm{Fe}}}}}},{{{{{\rm{Co}}}}}},{{{{{\rm{Ni}}}}}}\right)}_{2}}$, ${\left(002\right)}_{{{{{{\rm{A}}}}}}{{{{{{\rm{l}}}}}}}_{3}{{{{{\rm{Ti}}}}}}}$ // ${(001)}_{{{{{{\rm{A}}}}}}{{{{{{\rm{l}}}}}}}_{9}{\left({{{{{\rm{Fe}}}}}},{{{{{\rm{Co}}}}}},{{{{{\rm{Ni}}}}}}\right)}_{2}}$, ${\left(310\right)}_{{{{{{\rm{A}}}}}}{{{{{{\rm{l}}}}}}}_{3}{{{{{\rm{Ti}}}}}}}$ // ${(010)}_{{{{{{\rm{A}}}}}}{{{{{{\rm{l}}}}}}}_{9}{\left({{{{{\rm{Fe}}}}}},{{{{{\rm{Co}}}}}},{{{{{\rm{Ni}}}}}}\right)}_{2}}$ and their interplanar spacing has the following match ratio: ${{{{{{\rm{d}}}}}}}_{{{{{{\rm{A}}}}}}{{{{{{\rm{l}}}}}}}_{3}{{{{{\rm{Ti}}}}}}}^{(002)}:{{{{{{\rm{d}}}}}}}_{{{{{{\rm{A}}}}}}{{{{{{\rm{l}}}}}}}_{9}({{{{{\rm{Fe}}}}}},{{{{{\rm{Co}}}}}},{{{{{\rm{Ni}}}}}})_{2}}^{(001)}\,\approx$ 1:2 and ${{{{{{\rm{d}}}}}}}_{{{{{{\rm{A}}}}}}{{{{{{\rm{l}}}}}}}_{3}{{{{{\rm{Ti}}}}}}}^{(310)}:{{{{{{\rm{d}}}}}}}_{{{{{{\rm{A}}}}}}{{{{{{\rm{l}}}}}}}_{9}({{{{{\rm{Fe}}}}}},{{{{{\rm{Co}}}}}},{{{{{\rm{Ni}}}}}})_{2}}^{(010)} \approx$ 1:5. The lattice mismatch values estimated from diffraction patterns for these matching planes are 2.5% and 2.1%, respectively.

**Fig. 4:**
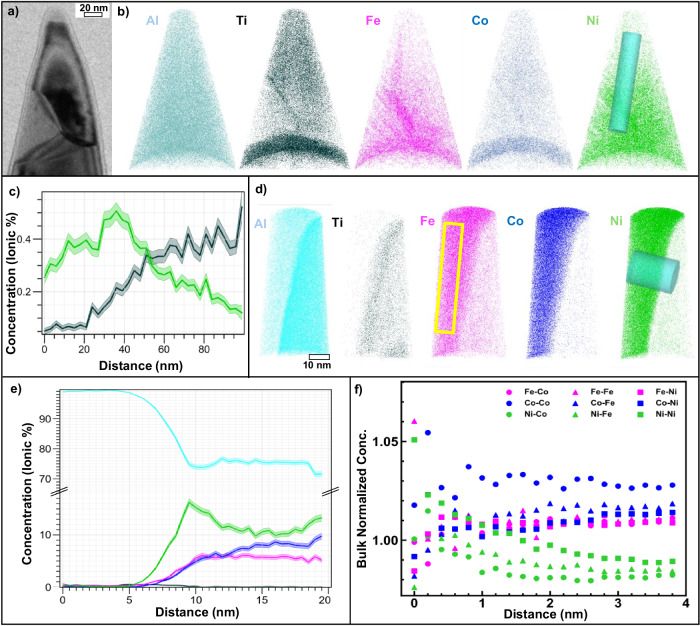
Atom Probe Tomography (APT) analyses. **a** An image of atom probe tip taken prior to APT acquisition, demonstrating a three-grain region. **b** Atomic maps demonstrating uneven saturation of solute elements. **c** 1D concentration profile (obtained from bottom to top of the cylindrical region of interest on Ni map in (**b**) capturing the behavior of the solute elements across two grains. The concentrations of Ti, Fe, Co, and Ni in these three Al grains are below 1 at.%. Notice also in (**c**) some segregation at grain boundaries, as well as the variability of solute concentration inside the different grains, e.g., Ni and Ti. Error bars display standard error for plotted concentrations from the sampled region of interest. **d** Atomic maps of a sample demonstrating an Al grain saturated with all solute elements, and the monoclinic Al_9_(Fe,Co,Ni)_2_ phase. **e** 1D concentration profile taken across the interphase boundary following the cylindrical region on Ni map in (**d**). The profile highlights a transition from a dilute Al grain on the right side, to the Al_9_(Fe,Co,Ni)_2_ phase on the left. Error bars display standard error for plotted concentrations from the sampled region of interest. **f** Radial Distribution Function (RDF) taken from the yellow inset in (**d**) highlights the absence of solute clustering in the bulk monoclinic phase.

Figure 4a corresponds to a TEM image of three Al matrix grains from a fine rosette region to gauge the microstructure before atom probe acquisition. Atomic maps for individual elements (Fig. 4b) show that there are some inhomogeneities in the distribution of the TM solute elements. This variation is highlighted in the 1D composition distribution in Fig. 4c, where the profile along the cylinder in Fig. 4b. Ti concentration increases from 0.1 to 0.5 at% towards the top grain with a decrease in Ni concentration concomitantly. Grain boundaries appear to enrich in Fe, Co and Ni. In general, the transition metal elements have very low solid solubility in Al: Ti < 0.05 at%, Fe: < 0.04 at%, Co < 0.05 at%, Ni < 0.04 at%. Hence the APT composition analysis in Fig. 4c suggests that Ni and Ti concentrations in Al (0.1–0.6 at.%), have largely exceeded their equilibrium solid solubility, presumably due to supersaturation from the rapid solidification process. Figure 4d corresponds to a dual-phase interface between Al and Al_9_(Fe,Co,Ni)_2_ taken from another area. A relatively small amount of TM solutes are present in the matrix, specifically, 0.20% Ti, 0.30% Fe, 0.03% Co and 0.09% Ni (at%). Ti is highly rejected by the intermetallic phase and present in the matrix. The 1D composition distribution profile in Fig. 4e along the cylinder shown in Fig. 4d shows minute TM solutes in matrix, vs. nearly equiatomic distribution of Fe, Co and Ni (78.06% Al, 0.06% Ti, 5.45% Fe, 7.53% Co and 8.90% Ni (at%)) in the intermetallic phase. To understand the interactions between the alloying elements in the brittle intermetallic region of the tip, partial radial distribution functions (Fig. 4f) were computed (in the area highlighted by the yellow rectangle). All profiles remain close to 1.0, the value for random distribution, signifying well-mixed TM solutes in lattice. Another tip on the Al_9_(Fe,Co,Ni)_2_/Al_3_Ti interface was examined by APT to confirm the chemical constituents of both intermetallic phases (Supplementary Fig. 4). There is no evidence of the presence of Al at the interfaces, suggesting rosettes are mostly intermetallics.

### Mechanical properties

**Fig. 5:**
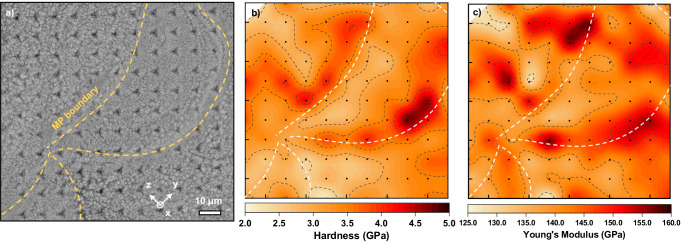
Hardness contours derived from nanoindentation results. **a** A back scattered scanning electron microscopy (SEM) micrograph on the as-printed Al_92_Ti_2_Fe_2_Co_2_Ni_2_ after nanoindentation hardness measurements. **b** The hardness and (**c**) Young’s modulus contour maps re-constructed from series of nanoindentation, revealing a relatively higher hardness/ Young’s modulus near the melt pool boundaries with finer microstructure.

In order to assess the mechanical properties of heterogenous AM Al alloys, nanoindentation experiments were conducted over a representative melt pool (Fig. 5a). Hardness contour reconstructed from nanoindentation map (Fig. 5b) shows a majority of the region has a high hardness ranging from 2.5 to 4.5 GPa. Melt pool boundaries often have a higher hardness (3.5–4.5 GPa), whereas the melt pool interior has a lower hardness (2.5–3.0 GPa). A similar trend for Young’s modulus is observed. Hard melt pool boundaries are associated with a relatively high Young’s modulus (140–150 GPa), while melt pool interiors have a relatively low Young’s modulus (130–140 GPa).

**Fig. 6:**
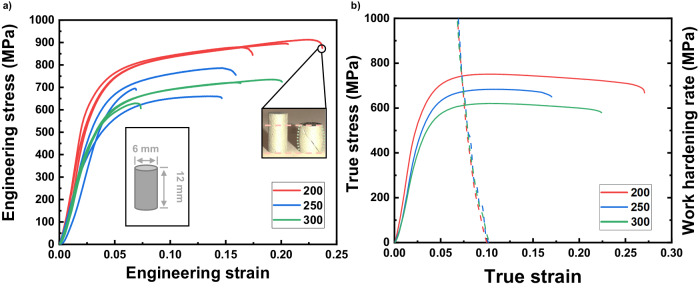
Macroscale mechanical properties under compression tests. **a** Engineering stress – engineering strain curves for bulk compression tests performed on the as-printed Al_92_Ti_2_Fe_2_Co_2_Ni_2_ pillars with inserted schematic diagrams showing the geometry of specimen and the typical barreling phenomenon after compression for one of the deformed specimens (200 W). Numbers in the legend denote laser power in Watts with three repetitions in each condition for reproducibility. **b** True stress - true strain curves (solid lines) superimposed with work hardening rates (dashed lines) showing 7% uniform compression strain in the early stages of deformation followed by long toughing stages.

Bulk compression tests were performed on cylinders with dimensions of 6 × 12 mm, fabricated at various laser power. Specimens printed with 200 W laser (red) exhibit an ultra-high engineering stress exceeding 800 MPa concurring with substantial plastic deformability around 20%. The inserted optical micrographs reflect the typical barreling phenomenon for ductile metallic materials. At higher laser energy, 250 W and 300 W, the flow stress of pillar decreases to 550 MPa with compressive strain of 5−20%. Figure 6b presents analyses on plastic instability by using Considère’s criterion. The superimpositions of selective work hardening curves over true stress – true strain curves imply uniform deformation strain at 7%.

**Fig. 7:**
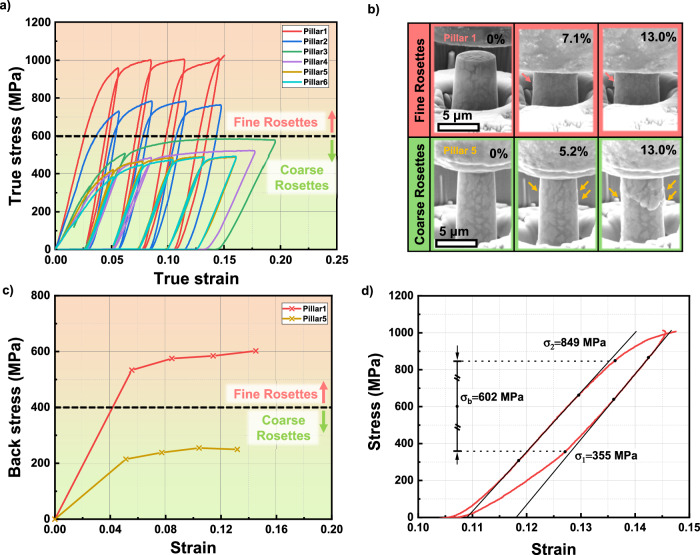
Microscale mechanical properties revealed by micropillar compression tests. **a** True stress – true strain curves for in situ micropillar compression tests on both fine (pink) and coarse (green) rosettes regions of samples printed with 300 W laser power tested at room temperature. The flow stress on fine rosette region could reach 1 GPa. **b** Screenshots of morphological evolutions for pillars under compression. Arrows indicate the formation of shear planes. **c** Back stress measurements vs. strain curves for micropillars with coarse and fine rosettes showing the prominent back stress. **d** An exemplar back stress determination based on a stress – strain hysteresis loop for the pillar in fine rosette region at 14% strain. Solid black curves based on elastic loading/unloading stages are derived from the linear sections and extrapolated to the yield points with 0.1% proof strain. The back stress value, ${\sigma }_{b}$, the yield stress for unloading, ${\sigma }_{1}$ and the yield stress for loading, ${\sigma }_{2}$ are labeled for illustration.

To probe the influence of heterogeneous microstructures on mechanical behavior of the AM Al alloys, micropillar compression tests were carried out over coarse and fine rosettes regions. As shown in Fig. 7a, fine rosette region can sometimes possess a high strength exceeding 900 MPa, while coarse rosette region has a flow stress of 500 MPa (Supplementary movies 1 and 2). In situ SEM micrographs in Fig. 7b collected from supplementary movies reveal the morphological evolution for representative pillars from these two distinct regions. Rosette precipitates readily visible on the pillar surfaces in the coarse rosette region due to axial compression, while a wrinkled and wavy surface shows less apparent protrusion in the fine rosette region. For the coarse rosettes, the evident rosette precipitate extrusions to the outer surfaces manifest the preferential plastic flow in the soft aluminum matrix and insignificant plasticity in the hard intermetallics precipitates. Whereas the retention of cylindrical shape of pillars in the fine rosettes implies uniform co-deformation of matrix and precipitates to constrain the generation of localized shear bands. The multiple loading-unloading experiments display prominent hysteresis loops evident from the stress-strain curves. The implication of such loops on deformation mechanisms will be discussed in the discussion section.

### Post-deformation microstructure analyses

**Fig. 8:**
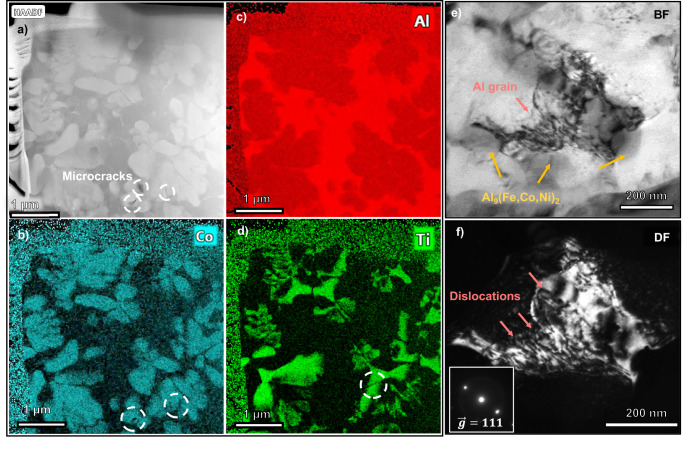
Deformation mechanisms in coarse rosette regions. **a**–**d** Scanning transmission electron microscopy (STEM) images and selective energy dispersive x-ray spectroscopy (EDS) mapping on the post-deformation pillar in the coarse rosette region. **e** BF and (**f**) weak-beam dark field TEM micrographs of the Al grain revealing high-density dislocations (denoted by red arrows) in the vicinity of cellular precipitates. The insert in (**f**) shows the diffraction spots after construction of weak beam condition g(3 g), g = 111.

To better understand the deformation mechanism of AM Al alloys with intermetallic rosettes, cross-section TEM (XTEM) samples from post-deformation micropillars were investigated. In the coarse rosette region (Fig. 8a), some microcracks are witnessed in intermetallics in STEM image. EDS elemental maps are provided in Fig. 8b–d to identify composition in these intermetallics precipitates. Microcracks (labeled in dash circles) appear in both Al_3_Ti and Al_9_(Fe,Co,Ni)_2_ intermetallics. It’s witnessed that these microcracks are typically constrained in single intermetallic phase, not extending into the neighboring phases. Figure 8e, f show abundant dislocation activities in the vicinity of cellular walls in Al matrix.

**Fig. 9:**
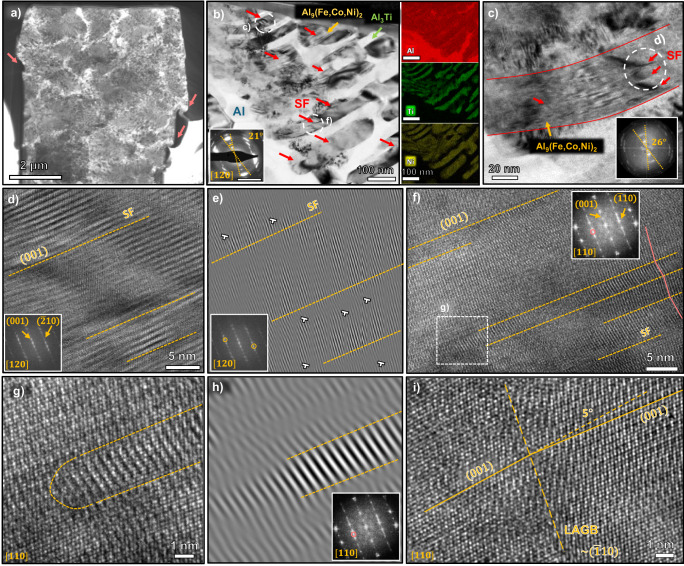
Deformation mechanisms in fine rosette regions. **a** An overview transmission electron microscopy (TEM) micrograph on the post-deformation pillar in the fine rosette region. Pink arrows indicate the deformation bands. **b** A TEM micrograph demonstrating the morphology of fine intermetallic rosettes. Phases are labeled (blue for Al, green arrows for Al_3_Ti and yellow arrows for Al_9_(Fe,Co,Ni)_2_) and energy dispersive x-ray spectroscopy (EDS) maps are presented. Red arrows indicate the abundant stacking faults (SFs) in the monoclinic Al_9_(Fe,Co,Ni)_2_. The insert shows the selected area electron diffraction (SAED) pattern along Al_9_(Fe,Co,Ni)_2_ [120] zone with the significant crystal rotation by plastic strain. The scale bar corresponds to 5 nm^−1^. **c** A TEM image for Al_9_(Fe,Co,Ni)_2_ outlined in red near the pillar top in (**b**) with the corresponding Fast Fourier Transform (FFT) pattern. Stacking faults and crystal rotation are observed. **d** An High-resolution TEM (HRTEM) micrograph for the Al_9_(Fe,Co,Ni)_2_ particle in (**c**) with planar defects. The corresponding FFT pattern reveals stacking faults habit plane is (001). **e** An inverse FFT image masking circled diffraction spots shows lattice distortion around stacking faults with some additional half planes, indicating the existence of dislocations. **f** An HRTEM micrograph for the Al_9_(Fe,Co,Ni)_2_ particle in (**b**) slightly off the pillar top with planar defects. The indexed FFT pattern along [110] zone shows diffraction spots originating from defects circled in pink. Pink solid line segments help to visualize the direction change of crystal planes. **g** An HRTEM micrograph on the end of a stacking fault ribbon. **h** An inverse FFT image masking the extra spots in the inserted FFT confirms they result from the stacking fault ribbon. **i** An HRTEM image for Al_9_(Fe,Co,Ni)_2_ showing a low-angle grain boundary (LAGB) ~ 5° close to $(1\bar{1}0)$ plane where atomic disorder is observed.

In the fine rosette region, the dilation of the pillar top and shear bands were observed (Fig. 9a). Apart from dislocation activities and microcracks, significant crystallographic rotation (as evidenced by the inserted SAED pattern) and some planar defects were observed in the monoclinic Al_9_(Fe,Co,Ni)_2_ phase (Fig. 9b). Figure 9c shows the Al_9_(Fe,Co,Ni)_2_ precipitate (outlined in red) has an evident curvature with planar defects and grain rotation on the pillar top, where the deformation is the most intense. The HRTEM micrograph of the Al_9_(Fe,Co,Ni)_2_ shows abundant stacking faults (Fig. 9d), and streaks in the inserted FFT pattern suggest the habit plane for these SFs is (001). The related inverse FFT image masking two brightest diffraction spots reveals dislocations aligned along the SFs (Fig. 9e). Another HRTEM micrograph (Fig. 9f) was acquired for Al_9_(Fe,Co,Ni)_2_ at 400 nm underneath the top surface, where the deformation is less severe. The lattice arrangement is disturbed by (001) SFs along [110] zone. Streaks in the indexed FFT pattern are pronounced and extra spots circled in pink are identified. Figure 9g depicts the ends of a SF ribbon. The corresponding inverse FFT image in Fig. 9h confirms the additional spots originate from the faulted region, which might suggest a possible phase transformation, as the interplanar spacing has no matching with the parent phase. Figure 9i demonstrates a low-angle grain boundary (~5°) within Al_9_(Fe,Co,Ni)_2_. The boundary is roughly on $(1\bar{1}0)$ where some local disorder is present.

## Discussion

A highly heterogeneous microstructure composed of coarse rosettes, fine rosettes and cellular Al matrix was observed in the AM Al alloy. To further understand the formation of rosettes, equilibrium phase constitution was calculated by Thermo-calc using TCAL8 database (Supplementary Fig. 5). The calculation suggests that Al_3_Ti forms the cores of intermetallic rosettes due to its high melting temperature, providing nucleation sites for co-precipitation of Al_9_(Fe,Co,Ni)_2_. The rapid cooling rate significantly refines precipitates, whereas traditional casting of transition-metal-bearing Al alloys often leads to overgrown large precipitates and hence, embrittlement. The morphology distinctions for coarse and fine rosettes are attributed to the complex and location-specific thermal history with respect to melt pools. It is postulated sufficient supplies of TM solutes and a higher quenching rate adjacent to melt pool boundaries enable the precipitation of a greater volume fraction of intermetallics with finer lamellae, compared to the coarse rosettes that dominate melt pool center. The arrangement of alternating fine and coarse rosettes region in this alloy results from periodic thermal cycles during layer-wise construction. Additional refinement is realized by the striated precipitation possibly due to the turbulent Marangoni flow^36^. Marangoni flow stirs fine rosettes owing to the complex thermal gradient, varying surface tension and dynamic hydromechanics^36^. Besides, the lattice matching between Al_3_Ti and Al_9_(Fe,Co,Ni)_2_ may reduce the interfacial energy, and promote the formation of nanolaminated intermetallics. Under the assumption of quasi-equilibrium condition, the crystal will exhibit anisotropic Wulff shapes to reduce interfacial energy, promoting the formation of refined intermetallic rosettes with larger interfacial area. The formation of high-melting-temperature intermetallics in the melt pools may promote heterogeneous nucleation of Al grains during subsequent solidification and thus reduce the grain size of Al. Similar effects have been reported for Al alloys with the aid of nucleants, such as Al_3_Zr^18–20^, Al_3_Sc^21,22^, Al_3_Ti^37^, TiB_2_^14–17^, for the refinement of grain size of Al matrix. The rosette structure was reported in other Al alloys where Ce and Mn were introduced for precipitate strengthening, yet the resultant deformation mechanisms remain unexplored^38–41^.

The high quenching rate characteristic of laser fusion not only refines the microstructure but also has profound impact on the formation of various non-equilibrium phases. First, L1_2_ Al_3_Ti is often unstable and will spontaneously transform to equilibrium D0_22_ Al_3_Ti^42^. But the rapid solidification process retains some L1_2_ Al_3_Ti (Supplementary Fig. 3) by not allowing atoms sufficient time for complete ordering. The cruciform geometry of Al_3_Ti core in Fig. 2 correlates well with literature reports on typical morphology characteristics of trialuminides^43,44^. L1_2_ Al_3_Ti can also be fabricated via mechanical alloying with or without a ternary element^30,45^. It is generally accepted that L1_2_ Al_3_Ti shall be more deformable than its D0_22_ counterpart as the former has more independent slip systems rendered by a cubic crystal structure. It is also speculated that the high cooling rate establishes significant defects in both phases. Second, a partitioned medium-entropy intermetallic phase Al_9_(Fe,Co,Ni)_2_ was maintained, while at equilibrium (Supplementary Fig. 5) it shall decompose into two isomorphic monoclinic phases (Al_9_Co_2_ + Al_9_FeNi). The supersaturated Fe, Ni atoms in Al_9_(Fe,Co,Ni)_2_ distort the lattice locally and thus change its mechanical behavior. Preservation of these metastable phases would play a significant role in deformation mechanisms of the AM Al alloys.

High strength of the current AM Al alloy is confirmed by multiple experiments. This alloy exhibits over 800 MPa engineering stress from macroscale compression tests. Micropillar compression tests show that the fine rosette region could reach 1 GPa true flow stress or an engineering stress of 1.18 GPa. An estimation based on the rule of mixture is attempted as shown in Supplementary Table S1^29,46,47^. Hardness assessments from nanoindentation mapping show values of 2.5–4.5 GPa. The variations of hardness values across melt pools arise from the heterogenous microstructures consisting of coarse rosettes in melt pool center and fine rosettes near melt pool boundaries. The current Al_92_Ti_2_Fe_2_Co_2_Ni_2_ has an excellent combination of mechanical strength and plastic strain under compression, compared with other AM Al alloys shown in Supplementary Fig. 6^24,48–51^.

Next, we consider the related strengthening mechanisms leading to the ultrahigh mechanical strength in our AM Al alloys, including solid solution strengthening and Orowan strengthening, Hall-Patch strengthening, dislocation strengthening, and hetero-deformation induced (HDI) strengthening^52^. Solid solution strengthening can be ignored as the accumulative solubility of TM solutes in Al (though in supersaturated state) is very low, <1 at% based on EDS measurements (Fig. 2) and APT studies (Fig. 4c). Dislocations in the as-printed state do not play a significant role in strengthening the alloy. TEM experiments (Supplementary Fig. 7) show a moderate dislocation density *ρ*_*disl*_ 4.7 × 10^13 ^m^−2^ ~ 1.0 × 10^14 ^m^−2^. Therefore, strengthening contribution from these dislocations can be estimated to be 25–36 MPa by:

1 $${\sigma }_{{disl}}={{{{{\rm{\beta }}}}}}{{{{{\rm{MGb}}}}}}\sqrt{{\rho }_{{disl}}}$$

where β is a material constant, M is the Taylor factor, G is shear modulus, b is the burgers vector of Al. Their values are given as follows, $\,{{{{{\rm{\beta }}}}}}=0.16$, ${{{{{\rm{M}}}}}}=3.06$, ${{{{{\rm{G}}}}}}=26.5\,{{{{{\rm{GPa}}}}}}$ and b = 0.286 nm^23,53^. Orowan strengthening is also secondary as the morphology of dislocations are not those typical to Orowan strengthening, which would be manifested by dislocation loops encircling intra-granular precipitates. In the current AM Al alloy, dislocation entanglements are primarily identified against inter-granular Al_9_(Fe,Co,Ni)_2_ cellular precipitates in the post-deformation TEM samples (Fig. 8e, f).

HDI strengthening has been observed in heterogenous materials and can provide back stress and work hardening ability in metallic materials^52,54,55^. Significant stress-strain hysteresis loops were observed during micropillar compression tests (Fig. 7a). The evolution of accumulated back stress with progressing strain in Fig. 7c reveals that the fine rosette regions with a higher flow strength carry a very high back stress, ~600 MPa, compared to the back stress of ~250 MPa in the coarse rosette regions. Back stress values, ${\sigma }_{b}$, are determined following the classic method^55^ and are demonstrated in Fig. 7d with the following equation:

2 $${\sigma }_{b}=\frac{{\sigma }_{1}+{\sigma }_{2}}{2}$$

where ${\sigma }_{1}$ and ${\sigma }_{2}$ are the yield stress values on the unloading stage and loading stage, respectively. Back stress is the long-range stress component typically ascribed to the pileups of geometrically necessary dislocations (GNDs) in materials with heterogeneity or gradient structures. These GNDs could arise from mismatch of coefficients of thermal expansion (CTE) between Al and intermetallics during solidification^56,57^, and strain incompatibility across interfaces of hard and soft phases during compression. It’s worth mentioning that HDI strengthening is the major component leading to Bauschinger effect as GND pileups near interfaces have reversible dislocation configuration during loading-unloading processes. TEM study reveals ample GNDs in Al matrix shown in Fig. 8e, f. Rigid intermetallics tend to remain elastically deformed while Al matrix sustains high-density dislocations near the interfaces to accommodate strain field gradient. Existing GNDs will hamper further dislocation motion and also trigger forward stress in the hard intermetallic phases across interfaces. Back stress and forward stress will collectively harden the material. As the deformation progresses, back stress rises quickly and reaches a plateau for both regions. This trend can be explained by the absorption of GNDs by the interfaces after straining to a critical value^58^. At certain strain levels, dynamic generation and annihilation of GNDs reach an equilibrium state, leading to a saturated strengthening contribution from HDI stress. The strain gradient carried by absorbed GNDs, though decoupled from strengthening, will trigger Al-intermetallics interface debonding in microscale and eventually fracture at macroscale^58^.

Apart from HDI stress from Al-intermetallic interface, there might be HDI stress originating from the interfaces between two genres of intermetallics Al_9_(Fe,Co,Ni)_2_ and D0_22_-Al_3_Ti. Prior study suggests both intermetallics phases are brittle at room temperature^29,59^. This assertion is especially applicable to Al_9_(Fe,Co,Ni)_2_ inferred from its monoclinic crystal structure with low symmetry. However, abundant SFs and dislocations were observed in the deformed nanoscale monolithic Al_9_(Fe,Co,Ni)_2_ (Fig. 9), suggesting unique plastic deformation mechanism in the often brittle intermetallics. This study presents what may be the first experimental evidence of plasticity in monoclinic Al_9_(Fe,Co,Ni)_2_ medium entropy intermetallics. There are limited studies showing the formation of complex intermetallics in AM Al alloys^39–41,60,61^. Some prior studies also suggest that complex metallic compound could deform by introducing metadislocations^62,63^, which rarely exist in high symmetry metallic materials, and metadislocations have been reported in Al_13_Co_4_ with monoclinic crystal structure^64^. There are dislocations in Al_3_Ti in the as-printed state (Fig. 3a–c). Hence, we speculate that dislocations should also carry out plastic flow in Al_3_Ti to ensure co-deformation between the two types of intermetallics across the laminated intermetallics interfaces in the fine rosette region (Figs. 2a and 9). Deformability and deformation mechanisms for nanoscale sandwiched intermetallic branches could differ from their bulk counterpart, due to the discrepancy in scale and confined loading state, as corroborated by studies on laminated nanolayers^65–68^. Comparing to transient and reversible GND pileups adjacent to Al-intermetallic interfaces, temporary defects configuration existing in intermetallic-intermetallic interface could generate back stress as well. For nanolaminated intermetallics, strain transfer into the monolithic Al_9_(Fe,Co,Ni)_2_ could be very challenging, which necessitates a large back stress to drive defect activity. Under such a large back stress, SFs or other defects may be activated in intermetallic phases. Due to the limited plasticity of intermetallics, HDI stress stemming from intermetallic interfaces would increase rapidly during the initial loading process and remain saturated after plastic relaxation, which is consistent with experiment observations of back stress saturation for both regions (Fig. 7c).

Under the context of strength-ductility paradox for most metallic materials, some factors contribute to around 20% plasticity in these high-strength AM alloys as shown from both macropillar and micropillar compression tests. First, the Al matrix accommodates a majority of plastic strain as verified by dislocations in Al in the deformed pillars. Second, the back stress from heterogeneous interfaces sustains significant work hardening. As discussed earlier, SFs and other defects have been observed in deformed nanoscale intermetallics to accommodate plasticity under high stresses. Third, the interfaces between the two nanoscale intermetallic phases may have increased the fracture strength in the fine rosette region, so that plastic yielding can occur before fracture. The improved fracture toughness of intermetallic nanolaminates is witnessed by microcracks restrained within lamellae in coarse rosettes, as shown in Fig. 8. This crack inhibition effect will release local stress concentration and delay catastrophic fracture.

In summary, a custom-made Al_92_Ti_2_Fe_2_Co_2_Ni_2_ alloy was fabricated by LPBF. This alloy has rosettes of nanoscale intermetallics and a macroscopic engineering compressive strength exceeding 800 MPa and 20% plasticity. Micropillar compression tests reveal that the fine rosette regions can achieve a microscopic compressive strength of nearly 1.0 GPa and at least 15% plasticity. The simultaneous achievement of high strength and plasticity arises from the large back stress accommodated through heterogenous intermetallic nanolaminate interfaces. Significant plasticity was also observed in the medium entropy monoclinic Al_9_(Fe,Co,Ni)_2_ intermetallic phases. The mechanisms that trigger the formation of abundant stacking faults in monolithic Al_9_(Fe,Co,Ni)_2_ remain to be illuminated by future modeling investigations. Our results shed light on incorporation of nanoscale intermetallics rosettes in the design of ultra-strong Al alloys with prominent plasticity.

## Methods

### Powder processing and manufacturing

Spherical powder with a nominal composition of Al_92_Ti_2_Fe_2_Co_2_Ni_2_ (at.%) satisfying −53 + 15 µm were gas atomized by Atlantic Equipment Engineering, Inc. Additive manufacturing was performed by using a laser powder bed fusion (LPBF) instrument, SLM 125 HL metal 3D printer in Argon atmosphere with the oxygen level below 1000 PPM. Printing was conducted by utilizing a 400 W IPG fiber laser (λ = 1070 nm) with a laser power of 200–300 W, a scan speed of 1200 mm/s, a hatch space of 100 µm, a layer thickness of 30 µm and a laser spot of 70 µm in diameter. Build plate was preheated to 200 °C and each layer rotated by 67°. Cylindrical samples with height 12 mm and diameter 6 mm were fabricated for bulk compression tests. Cubic samples with dimensions 10 × 10 × 5 mm were printed for microstructure characterization, nanoindentation and micropillar compression tests.

### Structural characterization

The microstructure of Al alloy was investigated by X-ray diffraction (XRD), scanning electron microscopy (SEM), transmission electron microscopy (TEM) and atom probe tomography (APT). Samples were mechanically grinded and polished down to 1 µm diamond paste. XRD was performed on a PANalytical Empyrean X’pert PRO MRD diffractometer with a 2 × Ge (220) hybrid monochromator to select Cu K_**α1**_ in the *2θ-ω* geometrical configuration. Scanning electron microscopy (SEM) experiments were performed by using a Thermo Fischer Quanta™ 3D and Teneo™ high-resolution Field Emission SEM microscopes with a back scattering detector operated at 30 kV. A Thermo Fisher Talos 200X TEM microscope with an acceleration voltage of 200 kV was utilized to capture bright field (BF), dark field (DF), scanning transmission electron microscopy (STEM) images, and Energy dispersive spectrometry (EDS) maps. Crystal orientation mapping was performed by using a NanoMEGAS detector. APT Samples were prepared using standard focused ion beam (FIB) lift-out procedures on a Scios 2 DualBeam FIB/SEM, followed by a series of annular milling steps with decreasing radii to achieve a tip radius of approximately 50 nm. Atom probe data were collected on a CAMECA LEAP 5000XS APT, using both voltage and laser mode acquisition. For the former, a pulse fraction of 20%, temperature of 50 K, and a pulse rate of 200 kHZ were employed. For the latter, similar values for temperature and pulse rate were employed, with a laser pulse energy of 80 pJ to ensure complete field ion evaporation. Data reconstruction and analyses were conducted using AP Suite 6.1 software.

### Mechanical testing

Nanoindentation experiments were performed with a Bruker’s Hysitron TI Premier nanoindenter with a Berkovich tip under displacement-control mode at 800 nm depth on well-polished samples. Hardness information was assessed from an area of 100 × 100 µm^2^ covering representative microscale features with 121 indents with 10 µm spacing in both dimensions. Progressive indentation with multiple continuous loading-unloading segments at incremental penetration depths were conducted for each indentation. Hardness and Young’s modulus were determined from an average of 10 measurements. Bulk compression tests were performed on an MTS framework with a 30 kN load cell and a strain rate of 10^−3 ^s^−1^ after polishing and leveling the top and bottom surfaces of as-printed cylindrical samples for better alignment. In situ micropillar compression tests were performed in the Quanta™ 3D SEM microscope equipped with a Hysitron PI 88× R PicoIndenter and a real-time video recorder. Micropillars were produced by FIB, with the height of 10 µm,, the diameter of 5 µm, and an aspect ratio of 2:1. Both 10 and 20 µm diamond flat-punch tips were used and strain rate was set as 5 × 10^−3 ^s^−1^. An average drift rate of 0.2–0.6 nm/s was determined for displacement correction.

### Supplementary information

Supplementary Information

Peer Review File

Description of Additional Supplementary Information

Supplementary Movie 1

Supplementary Movie 2

## Data Availability

The data supporting the findings of this study are available within the article and its supplementary Information. Additional data are available from the corresponding author on requests.
