# Supplementary material for: Additive manufacturing of an ultrastrong, deformable Al alloy with nanoscale intermetallics
Source: Nat Commun. 2024 Jun 15;15:5122. doi: 10.1038/s41467-024-48693-4 (PMC11180184; doi:10.1038/s41467-024-48693-4)
Supplement: Supplementary file 1 — Supplementary Information [file 41467_2024_48693_MOESM1_ESM.docx]

**Supplementary information for Additive manufacturing of an ultrastrong, deformable Al alloy with nanoscale intermetallics**

Anyu Shang^1^, Benjamin Stegman^1^, Kenyi Choy^2^, Tongjun Niu^1,3^, Chao Shen^1^, Zhongxia Shang^1^, Xuanyu Sheng^1^, Jack Lopez^1^, Luke Hoppenrath^1^, Bohua Peter Zhang^1^, Haiyan Wang^1^, Pascal Bellon^2^ and Xinghang Zhang^1^*

^1^School of Materials Engineering, Purdue University, West Lafayette, IN 47907, USA. ^2^Department of Materials Science and Engineering, University of Illinois Urbana-Champaign, Champaign, IL 61801, USA. ^3^Los Alamos National Lab, Albuquerque, NM 87545, USA. *email: xzhang98@purdue.edu

**Supplementary Figures**

**Supplementary Fig. 1** **XRD profile of the as-printed Al_92_Ti_2_Fe_2_Co_2_Ni_2_ alloy (at a laser power of 300W).** Different symbols identify several phases.


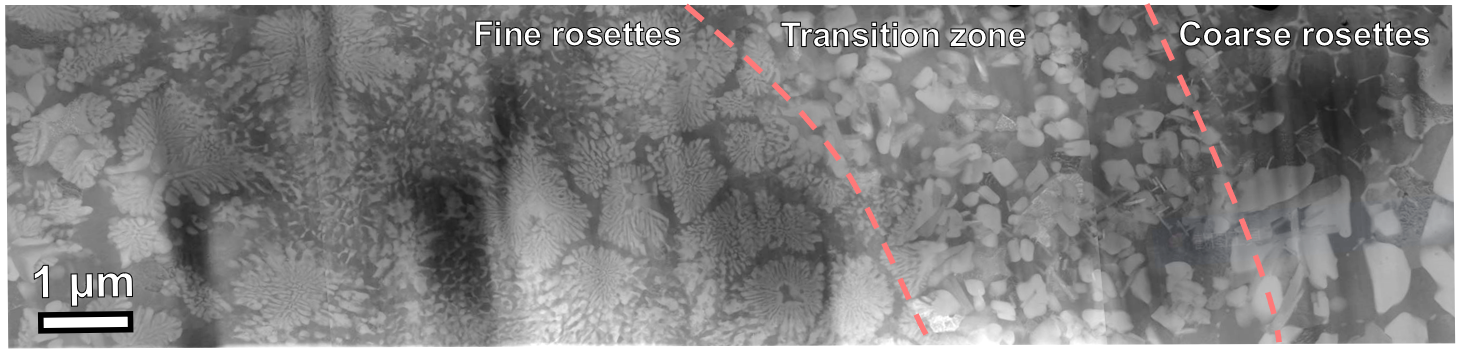


**Supplementary Fig. 2** **A TEM Panoramic view of fine and coarse rosettes regions showing the evolution of phases near melt pool boundaries.** The transition zone between fine and coarse rosettes regions has equiaxed precipitates.


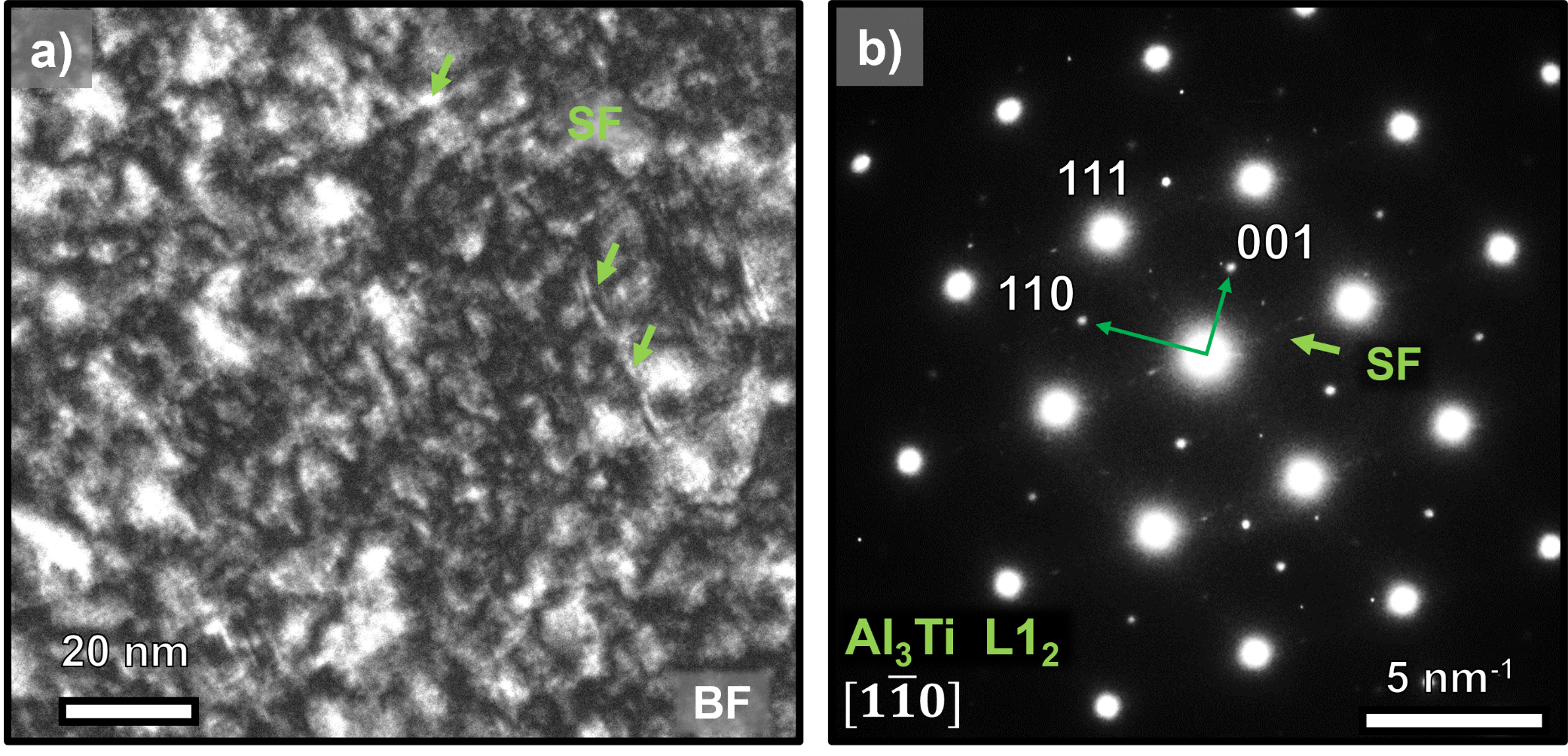


**Supplementary Fig. 3** **TEM micrographs of L1_2_ type Al_3_Ti (a) and its corresponding SAED pattern (b).** Lattice is also highly distorted and stacking faults (SF) are present.


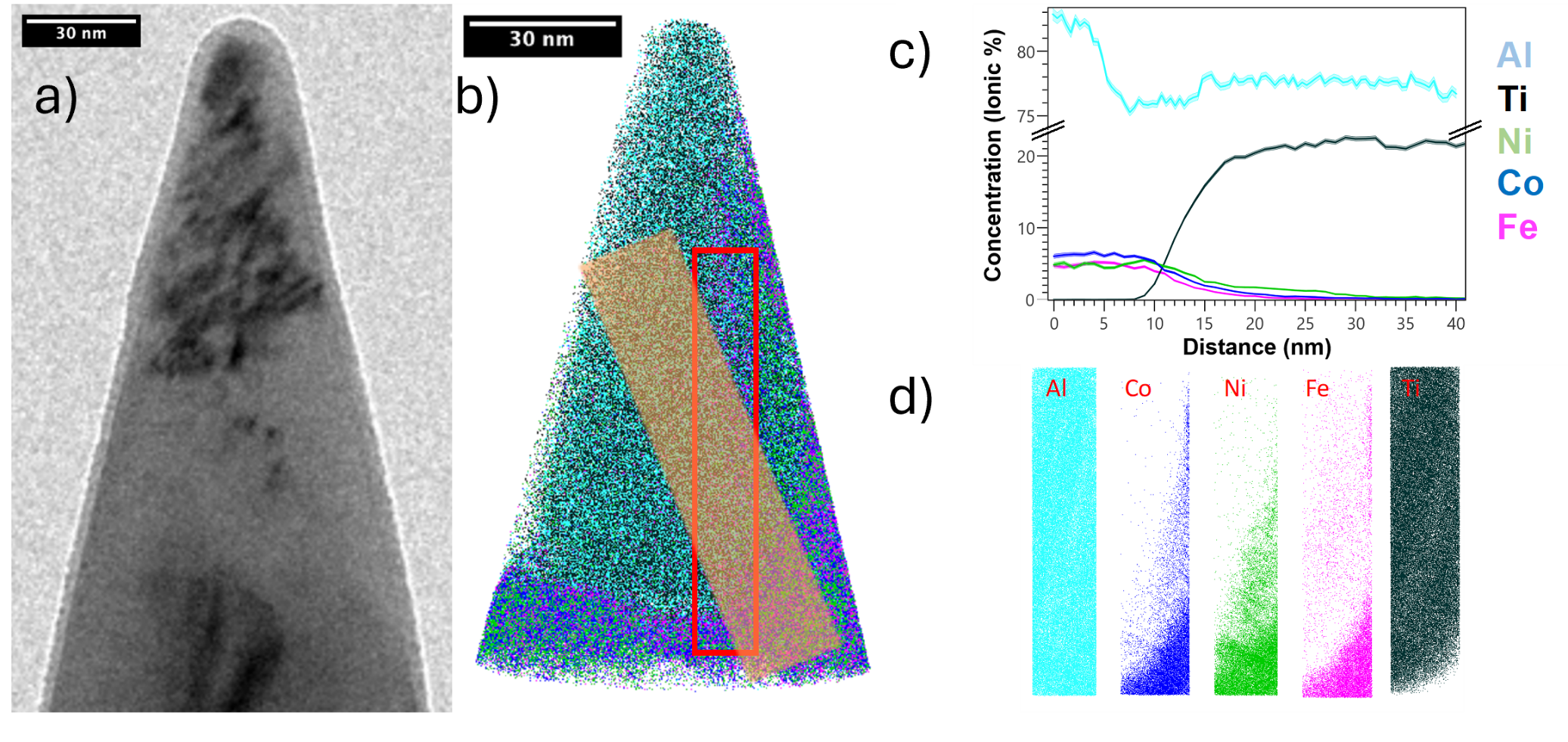


**Supplementary Fig. 4** **An APT composition analysis on the interphase interface between** **Al_3_Ti and Al_9_(Fe,Co,Ni)_2_.** a) Atom probe tip viewed under TEM at 200 kV prior to APT acquisition. b) Reconstruction of the atom probe tip acquired with laser mode. c) Concentration profile (obtained from bottom to top of the cuboidal region of interest in b) of constituent phases obtained in this tip. An Al_3_Ti phase dominates while an Al_9_(Fe,Co,Ni)_2_ phase is obtained in smaller proportions. Error bars display standard error for plotted concentrations from the sampled region of interest. d) Atomic maps of a selected region of interest demonstrating the interphase between Al_3_Ti and Al_9_(Fe,Co,Ni)_2_. The maps correspond to the red rectangular inset in b).

**
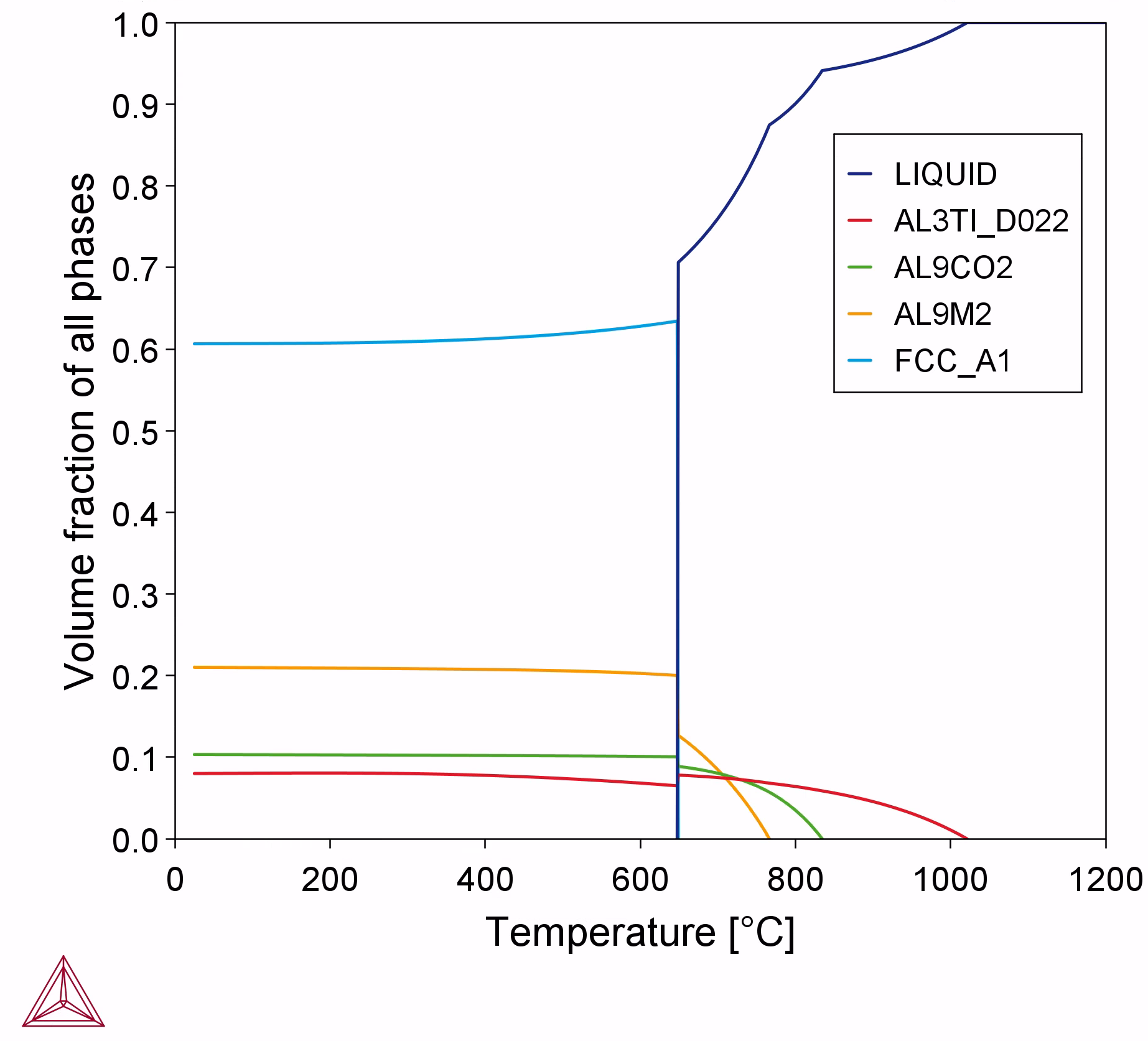
**

**Supplementary Fig. 5** **Thermo-calc results for equilibrium phases during solidification.** The calculation only enables relevant phases. “Al9M2” has a composition Al_9_(Fe,Ni)_2_.

**Supplementary Fig. 6**  **Peak engineering stress vs. compressive strain plot summarizing bulk mechanical properties of additively manufactured Al alloys under compression** ^1–5^**.** AlSi10Mg does not fracture at this strain. Micropillar compression experiments are terminated at 15%.


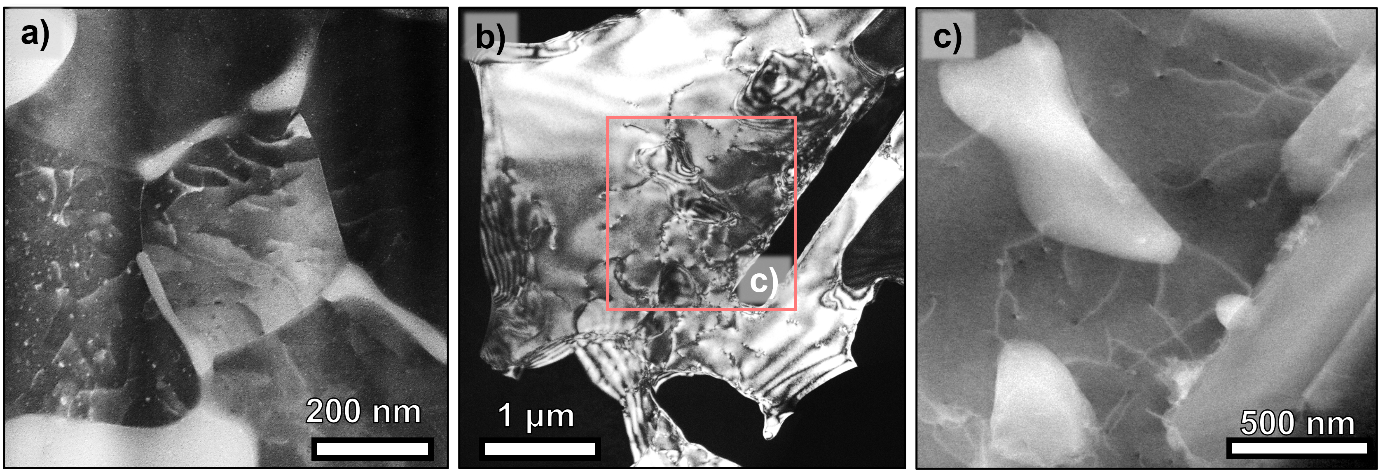


**Supplementary Fig. 7**  **Dislocation density estimation from TEM/STEM micrographs.** (a) STEM image on several cellular Al grains in Region 1. (b) TEM image on one Al grain in Region 2 and (c) the corresponding STEM image on the central area showing better contrast for dislocations.

**Supplementary Table 1** **A summary on the mechanical property data of the related phases** ^6–8^**.**

|  | Microhardness | Compression yield | Indentation Yield | Compression fracture |
| --- | --- | --- | --- | --- |
| Al | 235 MPa | 25 MPa | N/A | N/A |
| Al_3_Ti D0_22_ | 5.0 GPa | 354 MPa | 980 MPa | 354 MPa |
| (Al,Mn)_3_Ti L1_2_ | 2.0 GPa | 520 MPa | 570 MPa | 760 MPa |
| Al_9_(Fe,Co,Ni)_2_ | N/A | N/A | N/A | N/A |

**Supplementary References**

1. de Araujo, A. P. M. *et al.* Additive manufacturing of a quasicrystal-forming Al95Fe2Cr2Ti1 alloy with remarkable high-temperature strength and ductility. *Additive Manufacturing* **41**, 101960 (2021).

2. Wang, W., Takata, N., Suzuki, A., Kobashi, M. & Kato, M. High-temperature strength sustained by nano-sized eutectic structure of Al–Fe alloy manufactured by laser powder bed fusion. *Materials Science and Engineering: A* **838**, 142782 (2022).

3. Prashanth, K. G. *et al.* Production of high strength Al85Nd8Ni5Co2 alloy by selective laser melting. *Additive Manufacturing* **6**, 1–5 (2015).

4. Wang, P. *et al.* Microstructure and mechanical properties of Al-Cu alloys fabricated by selective laser melting of powder mixtures. *Journal of Alloys and Compounds* **735**, 2263–2266 (2018).

5. Aboulkhair, N. T., Maskery, I., Tuck, C., Ashcroft, I. & Everitt, N. M. The microstructure and mechanical properties of selectively laser melted AlSi10Mg: The effect of a conventional T6-like heat treatment. *Materials Science and Engineering: A* **667**, 139–146 (2016).

6. Milman, Yu. V. *et al.* Mechanical behaviour of Al3Ti intermetallic and L12 phases on its basis. *Intermetallics* **9**, 839–845 (2001).

7. Yamaguchi, M., Umakoshi, Y. & Yamane, T. Plastic deformation of the intermetallic compound Al3Ti. *Philosophical Magazine A* **55**, 301–315 (1987).

8. Takata, N., Takeyasu, S., Li, H., Suzuki, A. & Kobashi, M. Anomalous size-dependent strength in micropillar compression deformation of commercial-purity aluminum single-crystals. *Materials Science and Engineering: A* **772**, 138710 (2020).
