# Supplementary material for: Additive manufacturing of an ultrastrong, deformable Al alloy with nanoscale intermetallics
Source: Nat Commun. 2024 Jun 15;15:5122. doi: 10.1038/s41467-024-48693-4 (PMC11180184; doi:10.1038/s41467-024-48693-4)
Supplement: Supplementary file 3 — Description of Additional Supplementary Information [file 41467_2024_48693_MOESM3_ESM.pdf]

Description of Additional Supplementary Information

Supplementary Movie 1 In situ SEM video of a micropillar compression of coarse rosette region

Supplementary Movie 2 In situ SEM video of amicropillar compression of fine rosette region
